# Maternal Recreational Exercise during Pregnancy in relation to Children's BMI at 7 Years of Age

**DOI:** 10.1155/2012/920583

**Published:** 2012-04-03

**Authors:** Camilla Schou Andersen, Mette Juhl, Michael Gamborg, Thorkild I. A. Sørensen, Ellen Aagaard Nohr

**Affiliations:** ^1^Institute of Preventive Medicine, Copenhagen University Hospital, Øster Søgade 18,1, 1357 Copenhagen K, Denmark; ^2^Department of Public Health, Copenhagen University, 1014 Copenhagen, Denmark; ^3^Section of Epidemiology, Institute of Public Health, University of Aarhus, 8000 Aarhus C, Denmark

## Abstract

Exposures during fetal life may have long-term health consequences including risk of childhood overweight. We investigated the associations between maternal recreational exercise during early and late pregnancy and the children's body mass index (BMI) and risk of overweight at 7 years. Data on 40,280 mother-child pairs from the Danish National Birth Cohort was used. Self-reported information about exercise was obtained from telephone interviews around gestational weeks 16 and 30. Children's weight and height were reported in a 7-year follow-up and used to calculate BMI and overweight status. Data was analyzed using multiple linear and logistic regression models. Recreational exercise across pregnancy was inversely related to children's BMI and risk of overweight, but all associations were mainly explained by smoking habits, socioeconomic status, and maternal pre-pregnancy BMI. Additionally, we did not find exercise intensity or changes in exercise habits in pregnancy related to the children's BMI or risk of overweight.

## 1. Introduction

Keeping the high prevalence of childhood overweight in mind [1], it is imperative to look for all possible causes for this serious public health issue. It is well established that some intrauterine factors may influence later body size independent of birth weight [2–5]. Many maternal factors during pregnancy are potentially modifiable and therefore obvious targets for specific recommendations of beneficial behaviours during pregnancy to prevent childhood overweight.

Physical exercise in early pregnancy is related to maternal hormonal changes and placental modifications [6–10], leading to for example, altered substrate availability to the fetus [6, 8, 11–13] and lower levels of IGF-I and IGF-II concentrations in cord blood [14]. Hence, changes in the intrauterine environment due to maternal exercise may induce structural or epigenetic changes in the fetal brain or metabolic organs involved in body weight regulation later in life.

Maternal exercise during pregnancy has been associated with lower birth weight and lower risk of both small for gestational age (SGA) and large for gestational age (LGA) in the offspring [8, 16–21]. High birth weight and LGA have consistently been found as risk factors for overweight in childhood [22, 23], and SGA may be related to later visceral obesity and the metabolic syndrome [5, 24]. If maternal exercise has a normalizing effect on birth weight, it may also be associated with a normalization of BMI and a lower risk of overweight in the child beyond what is mediated through birth weight.

Only few studies have investigated the association between intrauterine exposure to maternal exercise and the child's later body size [25]. In a small cohort, there was no differences in weight or body fat at 1 year of age in children of exercising women and non-exercising women [26]. In contrast, an earlier case-control study on pregnant women suggested an inverse association between maternal exercise and weight and fat mass in the child at 5 years of age [27].

We used data from a large birth cohort to investigate the associations between different measures of maternal recreational exercise during early and late pregnancy and the children's BMI and risk of overweight at 7 years of age, while accounting for several potential confounding factors.

## 2. Materials and Methods

The study was based on data from the Danish National Birth Cohort (DNBC) in which 100,418 pregnancies among a total of 92,274 women were enrolled during the years 1996 to 2002. The cohort is described in detail elsewhere (http://www.dnbc.dk/) [28, 29]. Briefly, the women were recruited from all over Denmark in the beginning of pregnancy by their general practitioner. The women were telephone-interviewed twice during pregnancy at approximately week 16 and week 30 (Interview 1 and Interview 2) and twice after pregnancy at approximately 6 months and 18 months postpartum (Interview 3 and Interview 4).

A follow-up study of the children has been conducted. During the month that the child turned 7 years of age, the parents were asked to fill in either a mailed or web-based questionnaire about the child's health and well-being, including the latest height and weight measures. Some parents reported old measurements and some responded several years after having received the questionnaire, which gave an age span of 4.0–9.1 years of the children. In the beginning of 2011, 53,854 parents had responded to the 7-year follow-up.

In this study, live-born singletons born at 37–43 weeks of gestation (*n* = 50, 387), with information on childhood weight and height at follow-up (*n* = 48, 312), measured within the age span 5–8.5 y (*n* = 48, 218) were included. Exclusion criteria were as follows: no maternal participation in Interview 1 (*n* = 1, 952); maternal type I diabetes, gestational diabetes or preeclampsia (*n* = 1, 402); missing data on exercise from interview 1 (*n* = 178); or more than 30 days between children's weight and height measurements (*n* =  2, 218). Furthermore, some women (*n* = 2, 188) participated with more than one pregnancy in the cohort and only the first enrolled child was included in the analyses to avoid dependent observations. These exclusions led to a sample of 40,280 individuals for analysis.

### 2.1. Exposure Measures

The main exposure variables included amount of recreational exercise performed in early and late pregnancy as reported in Interview 1 and 2 in the DNBC. The time point of the interviews varied, with a median value (5 and 95 percentiles) of gestational week 16 (11, 25) for Interview 1 and gestational week 31 (27, 37) for Interview 2.

In both interviews, the women were asked: (1) “Now that you are pregnant, do you engage in any kind of exercise?” When the answer was yes, the following questions were posed: (2) “What kind of exercise do you engage in?”; (3) “How many times a week do you engage in……(answer in question 2)?”; (4) “How many minutes at a time do you engage in…… (answer in question 2)?”; (5) “Do you engage in other types of exercise?” In case of a positive answer to question 5, all the above questions were asked again, until a negative response was given. The questions asked referred to exercise as structured and planned activities. In case of uncertainty about whether an activity should be defined as exercise, it was included if the woman confirmed that it made her sweaty or short of breath. If the woman answered “no” to question 1, no further questions were asked about other possible forms of physical activity, for example, occupational or commuting activity.

The total number of minutes per week spent on recreational exercise was calculated from the duration of each exercise type and categorized into hours per week (h/wk): 0 h/wk, >0–≤1 h/wk, >1–≤2 h/wk, >2–≤3 h/wk, >3–≤5 h/wk, >5 h/wk. This information was further used to group the women according to whether they were non-exercising (0 h/wk) or exercising (>0 h/wk) in their leisure time.

Thirteen predefined types of exercise were categorized into seven groups of preferred activities defined as the type of exercise performed more than 50% of the time: Low-impact activities (aerobics/gymnastics for pregnant women, dance, walking/hiking, yoga) were activities where one foot always had to be on the ground while for high-impact activities (jogging, ball games, racket sports), both feet could leave the ground simultaneously. Other categories included swimming, workout/fitness training, bicycling, horseback riding, and a non-classifiable category for the women who did not have a preferred type of exercise. No questions during the two pregnancy interviews were specifically concerned about if, for example, cycling or walking was a transportation form or part of a more structured exercise plan, neither were the women asked about exercise practices prior to pregnancy.

From Interview 1, we also obtained information about the mother's self-reported pre-pregnancy weight, height, age at conception, parity, smoking during pregnancy, and socioeconomic status. Height and pre-pregnancy weight were used to calculate pre-pregnancy BMI (kg/m^2^); parity was categorized as primiparous or multiparous and smoking during pregnancy as non-smokers, 1–10 cigarettes/day, 11+ cigarettes/day. Socio-economic status was based on education and occupation and categorized as low, middle, or high. Information about total gestational weight gain and duration of exclusive breastfeeding (0–13 wk, 14–21 wk, or ≥22 wk) was obtained from Interview 3. In Interview 4, the mothers reported weight and length of the children at 5 months and 12 months of age, measured by the general practitioner or the public health nurse. Gestational age at birth and birth weight was obtained from the National Birth Register.

### 2.2. Outcome Measures

The outcome variable was the children's BMI at 7 years of age (median age: 7.1 (5 and 95 percentiles: 6.5 and 7.4, resp.)), calculated from the parent-reported weight and height measures at follow-up. The weight and height was based on measurements made by the school doctor, public health nurse, general practitioner, or by another person (i.e., the parent). It was used both as a continuous and a dichotomized variable (normal weight or overweight), according to international standards with age- and sex-specific cut-off points [30]. These cut-off scores correspond to percentile levels equal to an adult BMI of ≥25 kg/m^2^. Ten percent of the children in this cohort were overweight and only one percent were obese. Therefore, overweight and obese children were analysed together and referred to as overweight children.

### 2.3. Statistical Analysis

The student's *t*-test and *χ* ^2^-test were used to examine any group differences between non-exercising and exercising mothers. Multiple linear regression analyses were used to study the association between hours of recreational exercise per week and the child's BMI at 7 years of age, as well as the association between type of exercise and the child's BMI. After log transformation due to a non-normal distribution, BMI was standardized into age- and sex-specific *z*-scores by subtracting the individuals value (*X*_*i*_) with the population mean value (*X*_*p*_) and dividing this by the standard deviation obtained from the cohort (*SD*):

(1) $$Z=\frac{X_{i}-X_{p}}{SD}.$$

This approach was chosen because BMI changes considerably during childhood due to growth, and there was a relatively broad age span between the youngest and the oldest child in this cohort. The age-specific reference values were calculated for intervals of half a year.

Restricted cubic spline models were fitted to examine trends in data, but no significant deviations from linearity were observed (all values for *P* > 0.1).

In crude analyses, we included the child's age at measurements. The following covariates were chosen *a priori* and further included in the adjusted models: maternal age; parity; pre-pregnancy BMI; smoking during pregnancy; socio-economic status. In additional analyses, we also included total gestational weight gain, birth weight, gestational age at birth, duration of breastfeeding, and weight at 5 and 12 months to examine if there was any mediating effect of these variables on the associations between maternal exercise and the child's BMI. The same covariates were used in supplementary multiple linear regression analyses, comparing the non-exercising and exercising women for both early and late pregnancy.

We investigated whether the association between exercise in one period of pregnancy (early or late) and childhood BMI depended on the amount of exercise in the other period of pregnancy (late or early, resp.). We also analysed possible effect modification by sex, maternal smoking, pre-pregnancy BMI, and total gestational weight gain.

We repeated the analyses on a subpopulation restricted to women who completed Interview 1 in weeks 12–20 and Interview 2 in weeks 25–32 (*n* = 19, 461). These cut-points were chosen as a compromise between having a reasonable time distance between the two interviews (a minimum of 4 weeks) and at the same time including as many children as possible.

To investigate if any influence of maternal exercise was present specifically at the high end of the BMI distribution, we performed multiple logistic regression analyses with overweight at 7 years (yes/no) as outcome, using the previously-mentioned adjustment strategy.

All statistical analyses were performed using Intercooled STATA 9.2 (StataCorp, TX).

## 3. Results

Women who did not engage in any kind of recreational exercise in early pregnancy were generally older, had a higher pre-pregnancy BMI, and put on more weight during pregnancy than women who engaged in exercise (Table 1). Furthermore, the non-exercising women were more often multiparous, from a lower social class, smokers, and they breastfed their children for a shorter period of time. Similar patterns were seen for women who did not engage in exercise late in pregnancy. Twenty percent of the women engaged in exercise in both early and late pregnancy, whereas almost half of the women were non-exercising in both periods; 19% of the women reported exercise engagement in early, but not in late pregnancy, and 12% the other way around.

Maternal smoking, pre-pregnancy BMI, total gestational weight gain, or the child's sex did not modify the association between maternal exercise and the child's BMI, nor did exercise in one period modify the association between exercise in the other period and the child's BMI (data not shown).

In crude analyses, the amount of recreational exercise in both early and late pregnancy was overall inversely related to the children's BMI (Table 2). However, all associations were weakened after adjustment and became statistically non-significant. As an example, a 7-year-old girl with an average BMI (15.7 kg/m^2^) for this population would have a 0.02 unit lower BMI, if her mother performed >3 to ≤5 hours of exercise per week in early pregnancy compared with a peer, whose mother was non-exercising. The attenuation of the estimates was mainly explained by adjustment for smoking habits during pregnancy, followed by socio-economic status, and maternal pre-pregnancy BMI. In additionally adjusted analyses, we included total gestational weight gain, birth weight, gestational age at birth, duration of breastfeeding, and the child's weight at 5 and 12 months, but no noteworthy changes on the estimates were observed. There were no consistent trends indicating that exercise in one of the time periods during pregnancy was more strongly associated with children's BMI (Table 2).

In adjusted analyses, no associations between preferred type of exercise and children's BMI were found. Also, results from the analyses, where all exercising mothers were grouped together and compared to non-exercising mothers, were almost identical to the results from the main analyses (data not shown).

The analyses of childhood overweight as an outcome yielded the same picture as for childhood BMI. Thus, the risk of overweight was significantly higher among the children of non-exercising mothers in crude analyses, but after adjustment for the chosen covariates, all odds ratios were attenuated and became statistically insignificant (data not shown).

## 4. Discussion

In this study, we observed associations between different measures of maternal recreational exercise during early and late pregnancy and children's BMI and risk of overweight at 7 years of age. Adjustments revealed, however, that these associations were explained by maternal lifestyle factors, mainly smoking habits during pregnancy followed by socio-economic status and maternal pre-pregnancy BMI. This indicates that childhood overweight does not have its ground in the aspect of maternal activity level during pregnancy, and therefore this study provided no basis for recommending changes in exercise habits during pregnancy with the aim of reducing the risk of overweight in the child.

Previous studies of exercise and offspring outcomes have various methodological problems, such as small sample sizes, inadequate control for putative confounders, or insufficient classifications of physical activity performance [19, 20, 25, 31, 32], which our study aimed at coping with. Our cohort included a large number of participants, we used different measures of exercise (amount and type) in both early and late pregnancy, and due to the detailed information on a large number of covariates, we could control for important confounders and also study possible mediating effects of relevant lifestyle-related variables. Our study has, however, also some limitations as discussed in the following.

We aimed to examine recreational exercise in early and late pregnancy, but the timespan of reported exercise was broad. Some women categorized as inactive at Interview 1 could have performed high intensity exercise in very early pregnancy or the opposite. Nevertheless, in subanalyses of women who participated in the interviews close to their scheduled time, we found similar trends as in the main analyses. More precise information about timing of exercise is needed to resolve this problem, but we assume that any misclassification of exercise is nondifferential due to the prospective nature of the cohort. Most of our information was self-reported including data on maternal exercise, which may imply differential misclassification or simply imprecise information. However, a recent study on 112 pregnant women, carried out within the Norwegian Mother and Child Cohort Study (MoBa), found that questions on maternal gestational exercise correlated well with measurements from motion sensors [33]. The questions in DNBC are similar to those used in the MoBa study. An earlier comparison between the self-reported maternal pre-pregnancy BMI in the DNBC and measurements performed by their own general practitioner at the first antenatal care visit were in good agreement [34]. In addition, we performed a validation study of the parent-reported weight and height of the children in a sub-sample of the cohort (*n* = 1, 122) and found no systematic bias (details can be provided on request).

The present investigation did not include occupational or other physical activity than structured exercise and hence the study could not assess the influence of total daily activity load. This is a limitation of the study, since women who are physically active at work may exclude exercise in their leisure time. Thus, non-exercising women in this cohort could actually be more overall active than the women classified as active which may introduce some misclassification. However, in the MoBa, physical activity at work, defined as being short of breath/sweating at work at least once a week, was strongly positive associated with regular recreational exercise [35]. Since our cohort is very similar to the Norwegian cohort, we believe this association to be present in our cohort as well so that the exercise reported in our study also reflects the general activity level.

Few other studies have investigated the association between maternal exercise in pregnancy and later body size in childhood. In agreement with our results, Clapp III et al. [26] found similar weight and amount of body fat in 1-year-old children of exercising and intermittently active mothers. On the other hand, a case-control study, conducted by Clapp III et al. [27], did not agree with our results. It included 40 physically active women, where half of the women voluntarily stopped all activity during pregnancy. It showed that weight-bearing gestational exercise for ≥3 times/week for >30 minutes/session at a moderate intensity, compared to inactivity, was associated with lower weight and amount of fat in 5-year-old children. Although the study, being relatively small, obviously needs replication, there may be other reasons for the different results. It has been suggested that any influence from maternal exercise during pregnancy on the offspring may depend on the volume of the activity [25]. In the case-control study, only weight-bearing exercise was included, whereas a mixture of non-weight-bearing and weight-bearing exercise was undertaken by the women in our study. However, in our analyses on preferred kind of exercise, the weight-bearing activities (all types except swimming, bicycling, and horse-back riding) were not significantly associated with the children's BMI or risk of overweight. Another reason for the disagreement could be that the case-control study included women who were physically active before pregnancy, which may modify the association between maternal gestational exercise and the offspring's birth weight [14, 36]. Thus, athletes continuing their sports activity in pregnancy had babies with smaller birth weight than sedentary women initiating weight-bearing exercise in pregnancy [17]. We did not have information about the women's activity level before pregnancy and were therefore not able to assess its possible association with children's BMI.

It could be hypothesized that physical activity before pregnancy may modify the maternal pre-pregnancy BMI and thereby could play a role for later body size of the child. However, contrary to expectations, studies so far have not shown evidence of physical activity being a determinant of subsequent changes in BMI, neither in children nor adults [37]. Also, women who quit smoking due to their pregnancy may be more prone to weight gain [38], which could affect both exercise habits and the children's BMI. However, including gestational weight gain, in our analyses, had no noteworthy effect on the estimates. Our result may reflect that recreational exercise during pregnancy is part of an overall healthy lifestyle that includes less smoking, higher socio-economic status, and lower pre-pregnancy BMI, which all are strongly related to the children's BMI [4, 39, 40]. Thus, the crude associations in this study probably mirrored that exercising mothers in general were healthier and more active also in the postnatal period compared to non-exercising mothers.

Maternal and fetoplacental adaptations in relation to exercise of the mother have been suggested to be dependent upon the gestational period in which the exercise is performed [13]. Thus, timing of exercise in pregnancy may be important for body size in the child [12, 27, 41–44]. When compared to non-active mothers, children of mothers engaging in high intensity activity in the beginning of pregnancy and low intensity in late pregnancy were heavier at birth, whereas children of mothers who had the opposite pattern of activity were lighter [12]. In the present study, the timing or intensity of exercise during pregnancy was not associated with childhood BMI. If any biological explanation exists for the observed inverse association between gestational exercise and birth weight, the influence may only be immediate on the birth outcome and do not appear to exert any long-term effect detectable in childhood.

Women in the DNBC are likely to be healthier in general than the background pregnant population [29]. Furthermore, children in the present study had mothers who were older, of higher socio-economic status, higher parity, lower pre-pregnancy BMI, and less likely to smoke compared with excluded children. However, we did not find that pre-pregnancy BMI, total gestational weight gain, or maternal smoking modified the associations between gestational exercise and childhood BMI. We therefore assume that any relation between gestational exercise and the children's body size is the same for both included and excluded individuals and also applies to other children of mothers having similar lifestyle behaviours living in affluent countries.

In conclusion, we found an inverse association between maternal recreational exercise during pregnancy and childhood BMI or risk of overweight that could be explained by other lifestyle factors that differed between exercising and non-exercising mothers.

##  Conflict of Interest

There was no conflict of interest.

## Figures and Tables

**Table 1:** Characteristics of mothers according to recreational exercise in early pregnancy^a^, Danish National Birth Cohort, 1996–2002.

	All mothers (*n* = 40,280)		Non-exercising mothers (*n* = 24,492)		Exercising mothers (*n* = 15,788)	
	Mean	SD	Mean	SD	Mean	SD
Maternal age (years)	30.6	4.2	30.8	4.2	30.4	4.1***
Pre-pregnancy body mass index (kg/m^2^)	23.3	3.9	23.4	4.0	23.2	3.8***
Gestational weight gain (kg)	15.3	5.3	15.4	5.5	15.1	5.0***
Children's birth weight (g)	3,649	490	3,655	493	3,640	486**
Children's gestational age (days)	281.6	8.8	281.5	8.8	281.7	8.8*
Children's age at follow-up	7.1	0.3	7.1	0.3	7.1	0.3
Body mass index at 7 years (kg/m^2^)	15.7	1.7	15.7	1.7	15.6	1.6***

	No.	%	No.	%	No.	%

Parity						
Primiparous	19,436	48%	10,186	42%	9,206	58%
Multiparous	20,889	52%	14,288	58%	6,571	42%
*P*-value for trend						0.01
Socio-economic status						
High	22,593	56%	12,761	52%	9,797	62%
Middle	14,565	36%	9,478	39%	5,053	32%
Low	3,077	8%	2,169	9%	905	6%
*P*-value for trend						0.01
Smoking						
Non-smokers	30,892	77%	18,074	74%	12,756	81%
0–9 cigarettes/day	7,402	18%	4,858	20%	2,532	16%
≥10 cigarettes/day	2,061	5%	1,560	6%	497	3%
*P*-value for trend						0.01
Breastfeeding						
None or <14 weeks	9,058	28%	5,869	29%	3,173	25%
14–21 weeks	14,368	44%	8,586	43%	5,762	45%
≥21 weeks	9,436	28%	5,627	28%	3,786	30%
*P*-value for trend						0.01

Data are presented as mean values ± standard deviation or number of individuals (%).

*P*  value using student's *t*-test or **χ** ^2^-test, *P-value < 0.05, **< 0.005, ***< 0.001 (*P*  value are 2-sided).

Abbreviations: SD, standard deviation.

^a^Missing data on pre-pregnancy BMI (*n* = 624), gestational weight gain (*n* = 8,050), birth weight (*n* = 223), parity (*n* = 30), socio-economic status (*n* = 120), and breastfeeding (*n* = 7,493).

**Table 2:** Associations between maternal recreational exercise during pregnancy and the children's, body mass index at 7 years of age (*z*-scores of log-body mass index and differences in body mass index units), Danish National Birth Cohort, 1996–2002.

	Log-body mass index z-scores					Differences in body mass index units^c^	
Exercise							
(hours/week)					*P*-value		
Early pregnancy	No.	Crude^a^	Adjusted^b^	95% CI	for trend		

0	24,492	0	0	0		0	0
>0 to ≤1	5,707	−0.038*	−0.004	−0.032, 0.024		−0.02	−0.06, 0.05
>1 to ≤2	4,432	−0.065**	−0.021	−0.052, 0.010		−0.03	−0.09, 0.02
>2 to ≤3	2,377	−0.034	0.012	−0.029, 0.053		0.02	−0.05, 0.09
>3 to ≤5	2,152	−0.081**	−0.009	−0.051, 0.034		−0.02	−0.08, 0.06
>5	1,120	0.030	0.036	−0.022, 0.094	0.54	0.06	−0.03, 0.16
Exercise							
(hours/week)							
Late pregnancy							

0	26,149	0	0	0		0	0
>0 to ≤1	5,942	−0.059**	−0.013	−0.040, 0.0l5		−0.02	−0.06, 0.03
>1 to ≤2	3,288	−0.038*	0.020	−0.015, 0.056		0.03	−0.03, 0.09
>2 to ≤3	1,386	−0.101**	−0.030	−0.082, 0.023		−0.05	−0.14, 0.03
>3 to ≤5	1,128	−0.056	0.018	−0.041,0.076		0.03	−0.06, 0.13
>5	598	−0.114**	−0.047	−0.126, 0.032	0.36	−0.08	−0.22, 0.06

CI: confidence interval.

*P < 0.05, **P < 0.01 (*P* values are 2-sided).

^a^Crude models adjusted for children's age at follow-up.

^b^Additionally adjusted for maternal age, parity, pre-pregnancy body mass index, smoking and socio-economic status.

^c^A djusted changes in body mass index units in a 7-year old girl with mean body mass index of 15.67 if the mother changes exercise habits from inactive to one of the repective active groups.
